# Autobiographical memory and hierarchical search strategies in depressed and non-depressed participants

**DOI:** 10.1186/s12888-014-0310-z

**Published:** 2014-11-18

**Authors:** Shamsul Haque, Eka Juliana, Rahmattullah Khan, Penelope Hasking

**Affiliations:** Jeffrey Cheah School of Medicine and Health Sciences, Monash University Malaysia, Jalan Lagoon Selatan, Bandar Sunway, 47500 Subang Jaya, Selangor Malaysia; Department of Psychology, International Islamic University Malaysia, Jalan Gombak, Kuala Lumpur, Selangor Malaysia; Department of Psychology & Counseling, Sultan Idris Education University, Tanjong Malim, Perak Malaysia; School of Psychology & Speech Pathology, Curtin University, Perth, Western Australia 6845 Australia

**Keywords:** Autobiographical memory, Self-memory system, Depression, Hierarchical search, Autobiographical knowledge-base

## Abstract

**Background:**

There is a growing body of literature showing individuals with depression and other trauma-related disorders (e.g., posttraumatic stress disorder) recall more overgeneral and less specific autobiographical memories compared to normal participants. Although the mechanisms underlying overgeneral memory are quite clear, the search strategy operated within the autobiographical knowledge base, at time of recollection, requires further exploration. The current study aimed to examine the hierarchical search sequence used to recall autobiographical memories in depressed and non-depressed participants, with a view to determining whether depressed participants exhibited truncated search strategies.

**Methods:**

Thirteen depressed and an equal number of non-depressed participants retrieved 15 memories each, in response to 15 commonly used cue words. Participants reported the first memory that entered in their mind. All memory descriptions were recorded and later transcribed verbatim for content analysis.

**Results:**

Depressed participants retrieved autobiographical memories faster, produced shorter memory descriptions and were less likely to recall positive memories than non-depressed participants. Non-depressed participants were more likely to commence retrieval by accessing lifetime period knowledge followed by general event and event specific knowledge, whereas depressed participants showed a tendency to terminate retrieval at the general event level.

**Conclusions:**

It is concluded that depressed participants do adhere to the same hierarchical search strategy as non-depressed participants when retrieving specific autobiographical memories, but that they terminate their search early, resulting in overgeneral memories.

## Background

Since first reported by Williams and Broadbent [1], research interest has focused on examining memory retrieval in people with depression, who have consistently demonstrated a tendency to recall more general, and less specific autobiographical memories [2-5]. Specifically, overgeneral recall has been related to failure to recover from depression [6], severity of depression [7], and is over-represented in suicide attempters relative to control participants [8,1], even when controlling for depression [9]. This pattern of recollection is consistent across studies irrespective of the methods used to initiate the retrieval process (i.e. free recall or cued recall), and has been observed in adults, college students [10] and children [11]. Research indicates that overgeneral memory (OGM) is a vulnerability factor for depression and posttraumatic stress disorder (PTSD). It predicts the onset and/or recurrence of depression and PTSD [12,13] and a worse course of depression ([14,15] for a meta-analytic review). In early studies, it was also revealed deficiencies in social problem solving [16] and feelings of increased hopelessness [17] are associated with OGM.

In considering an explanation for these findings, the majority of researchers rely on the view of memory retrieval proposed by Conway and Pleydell-Pearce [18]. Conway and Pleydell-Pearce’s model, which they called *Self-Memory System* (SMS), expanded on earlier proposals that the retrieval of autobiographical memory is a relatively lengthy process (usually 5s-10s) which repeats through cycles of knowledge elaboration, access, and evaluation [19,20]. The SMS is a superordinate memory system in which three sub-ordinate systems (working self, retrieval models, and knowledge base) are coordinated in a task to construct a memory. When an individual is requested to retrieve an autobiographical memory in response to a cue word, a *retrieval model* is first created against which knowledge is accessed, evaluated and elaborated for further search. The *working self* that contains currently active goals and plans of the self operates to create this *retrieval model*. The SMS is also conceptualized as an *emergent* memory system as it occurs only when the working self interacts with the hierarchically organized knowledge base while in retrieval mode [21,22].

The current study is concerned with the hierarchical search within the autobiographical knowledge base that contains lifetime period knowledge, general event knowledge, and event specific knowledge [18]. *Lifetime periods* that form the highest level of hierarchy are the most general, most abstract, or most inclusive types of knowledge and denote time periods typically measured in units of years (e.g. *Living with ‘X’; It happened during our liberation struggle in 1971*). *General events* that form the middle level represent more specific types of event knowledge typically measured in units of months, weeks, and days. General events are normally composed of event memories which are either repeated or temporarily extended and thus lack temporal specificity (e.g. *first day at work, working in the office, suffering from tonsillitis*). In contrast, specific events, or *event specific knowledge*, which constitute the bottom level refer to memories of events that occur at one specific point in time and are typically measured in units of seconds, minutes or hours (e.g. …*one of them came very close to me, slapped my face and asked my name*).

It has been proposed that in individuals with depression, the failure to recall specific autobiographical memories is the result of a truncated search strategy - that is a failure of hierarchical search strategies whereby individuals retrieve information early in the hierarchy but fail to retrieve specific examples or events. Three hypotheses have been proposed that are summarized in the CaR-FA-X model to explain how this search may be truncated in depressed individuals: *capture and rumination* (CaR) hypothesis, *functional avoidance* (FA) hypothesis and *impaired executive control* (X) hypothesis [23]. More recent findings supporting this model have been thoroughly discussed by Sumner [24]. According to the impaired executive control hypothesis, retrieval processes require oversight by the central executive, and working memory capacity to initiate and maintain the search within the autobiographical knowledge structure [18]. Interfering with these processes, by distracting attention or overloading working memory, leads to early termination of search strategies and non-specific, overgeneral autobiographical memories [2].

The functional avoidance hypothesis suggests that people retrieve overgeneral memories as a method of avoiding negative affect [25]. Specifically, retrieval of detailed, especially negative, memories is thought to cause distress, and thus retrieval of overgeneral memories is negatively reinforced. Supporting this, retrieval of negative events has been found to produce less distress in those who tend to recall overgeneral memories, and less specific memory retrieval is associated with a repressive coping style [26].

Finally, capture and rumination, the tendency to dwell upon events and thoughts, is considered to be one reason for overgeneral memory retrieval [27], and may operate in two ways. First, in line with the central executive hypothesis, rumination may monopolise working memory capacity, limiting the availability of resources required to extract specific memories [23]. Secondly, people tend to ruminate about things that concern them. When asked to retrieve memories in response to cue words, these cue words map onto the current concerns of individuals rather than prompting a search for a new memory [28]. This mapping of cues and concerns results in retrieval of abstract, self-related knowledge rather than specific memories [29]. Consequently, rumination results in more recall of negative self-referent memories [30], while rumination about negative events has been shown to lead to more overgeneral memories than positive rumination [31].

Rather than working as separate processes, these three processes may work together to produce overgeneral autobiographical memory among people with depression. As limited attention and rumination are often present in depression it is probable that in a depressed individual, rumination facilitates mapping of cue words onto concerns of the individual and monopolises working memory capacity. Thus the cue word is mapped to the concern rather than activating a new search for a specific memory, and working memory capacity is compromised, limiting the resources available to conduct a search for a specific event. Coupled with a desire to avoid negative affect and limit intrusive memories, the search for specific memories tends to be terminated at an early stage in the search hierarchy.

Overgeneral recall has several consequences for depressed individuals. Most notably, overgeneral memories have been related to impaired problem solving ability and ability to generate solutions to potential future events [8,23], a concern given the emphasis of coping skills and problem solving in many psychological treatments for depression. However, although ample studies have investigated the specificity of autobiographical memory in depressed patients, and have examined the roles of rumination, affect regulation and attention in the relationship between overgeneral memory and depression, few studies have examined the sequencing of memory retrieval exhibited by depressed individuals, to determine whether they retrieve information in the hierarchical fashion proposed by Conway and Pleydell-Pearce [18]. Specifically, while non-clinical participants have been shown to access life time period knowledge, followed by general event knowledge and event specific knowledge when retrieving an autobiographical memory [32], it is unclear whether depressed individuals also access memories in this manner, or whether the search sequence differs. Understanding how depressed individuals access the knowledge hierarchy may aid in developing more effective methods of enhancing recall of specific memories, and of facilitating effective problem solving in this population.

The present study aimed to compare depressed and non-depressed participants in relation to retrieval of autobiographical memories. Specifically we expected that 1) depressed participants would retrieve more general and less specific autobiographical memories than non-depressed participants; 2) depressed participants would report more negative than positive memories relative to the non-depressed group; 3) depressed participants would terminate their retrieval search earlier in the search hierarchy than non-depressed participants.

## Methods

### Participants

Thirteen depressed patients (7 females, 6 males) and 13 non-depressed individuals (9 females, 4 males) participated in the study. The depressed group consisted of 8 out-patients and 5 in-patients at a large metropolitan hospital in Kuala Lumpur. Twenty five patients were initially invited to participate but 12 declined. All patients were diagnosed by a hospital psychiatrist as meeting the DSM-IV criteria for major depressive disorder [33]. The non-depressed participants were recruited through advertisements posted at a large university campus in Selangor, Malaysia, which invited interested individuals to contact the researchers if they wished to participate in a study regarding how people recall memories for personal events. The recruitment of non-depressed participants was made in such a manner that they were equivalent to the patients in terms of age and education. The average length of schooling was similar in depressed (M = 13.46 years, SD = 1.45) and non-depressed (13.85 years, SD = 1.52) participants, *t*(24) = 0.66, p = 0.52. The mean chronological ages were also comparable between depressed (36.00 years, SD = 11.97) and non-depressed (36.53 years, SD = 12.32) groups, *t*(24) = 0.113, p = 0.91. Three exclusion criteria were used for both groups; (i) age below 18, (ii) inability to speak Bahasa Malaysia (Malay Language), and (iii) present intoxication with drugs or other addictive substances.

### Materials

#### Autobiographical memory test by cue words

We utilised the cued-recall paradigm first used by Galton [34] and later adopted by Williams and Broadbent [1] in which memory retrieval was cued by words commonly used in everyday life. Fifteen words from five categories (common locations, general objects, positive emotions, negative emotions, and significant others) were chosen from lists of words used in previous autobiographical memory studies [35]. The words were: restaurant, beach, cinema (common locations); car, chair, telephone (general objects); happy, success, satisfaction (positive emotions); sad, guilty, regretful (negative emotions); and father, mother, friends (significant others). The words were administered in Bahasa Malaysia (Malaysian language), and presented in a random order to participants.

#### Beck depression inventory (BDI)

The BDI [36] consists of 21 forced choice items. Participants were asked to mark the items that best described how they felt the previous week. This test was given to the non-depressed participants to ensure that they were not showing any depressive symptoms. The participants scoring above 20 (indicating moderate depression) were excluded from participating in the non-depressed sample.

### Procedure

Use of data previously collected for a postgraduate degree, for the purposes of the current project, was approved by the Monash University Human Research Ethics Committee (CF13/2878 - 2013001547). The process of data collection started with the participants reading an explanatory statement. Participants were also verbally informed of the purpose of the study with an assurance that their participation would be voluntary and confidential, and the data they produce would be used for academic purposes only. Participants were cautioned that they may feel distressed while recalling some of their personal memories, but could withdraw from the study at any time. Interested participants signed a written consent form, prior to data collection.

All participants were tested individually by the second author. The patients were tested at the hospital, while the non-depressed group were tested at the university campus. After providing written consent, participants completed the Autobiographical Memory Test. Each participant was presented with one cue word at a time and asked to bring to mind a specific memory the word reminded them of. The participants were informed that a specific memory refers to a personally experienced event that happened at a particular time (within one day) and place, and were told the event could be important or trivial. They were also informed that memories could be retrieved from any point of their life, excluding the last month. Particular examples were given to clarify what the term “specific” means; retrieving information such as “*I jog every morning in the park”* in response to the cue word “*park*” would not be appropriate as it does not contain any specific time. However, a response such as “*Two days after we moved to our new house, I, along with my wife, went to the nearest park for a morning walk and surprisingly saw there one of my childhood friends approaching me and smiling*” would be suitable. In order to ensure participants were able to understand their tasks correctly, they completed two practice trials in which two neutral items (“bread” and “grass”) were used. If necessary, additional practice trials were arranged until the participants were successful in retrieving a specific memory.

In each trial, a cue word printed in capital letters on a white card was presented. When the participants had a memory in mind they were asked to verbally describe the memory; descriptions were tape-recorded. Participants were given one minute to retrieve a specific memory after each cue was presented. If participants did not recall a memory in the given time, the next cue was presented. A stopwatch was used to measure the retrieval time. Once a memory was retrieved there was no time limit on describing the memory. Total testing time for a participant (both depressed and non-depressed) averaged between one and one-and-a-half hours.

### Statistical analysis

The transcribed memory descriptions were examined for *elements* such as, lifetime period knowledge (LTP), general event knowledge (GE), event specific knowledge (ESK), thought (TH), mixed information (MIX), and other knowledge (OTR) ([37] for similar categorization). The main knowledge types (lifetime period, general event, event specific) were operationalised according to the definitions provided above. The *thoughts* category refers to the elements in which beliefs or conjectures were recalled, rather than specific or general information (e.g. *I feel I was the most stupid student in Cairo um…I mean in my class*). The *mixed* information category refers to the element in which various types of thoughts and information are recalled (e.g. *My father was pleased by such a gift because the person who presented it was actually his disciple and now became the administrative head of our district*). The *other* category refers to the elements in which no information associated with thought, lifetime period knowledge, general event, and event specific knowledge is reported. The coding for these six types of knowledge was completed by the second author and an external rater. A high level of consistency was observed (90% agreement), with disagreements resolved through discussion.

The memory descriptions were also examined for knowledge sequence as we wanted to see if the two groups varied in terms of overall construction of their autobiographical memories. For this purpose, memories were selected on the basis that they were described with some elaboration and contained any combination of LTP, GE and ESK. Memories in which the participants only reported TH, MIX and/or ORT knowledge were excluded. Each memory was coded according to the knowledge sequence (e.g., LTP > GE > ESK; LTP > ESK; LTP > GE; GE > ESK; and GE > LTP). To examine the consistency of coding, the second author and an external rater coded 30% of the memory protocols (95% agreement). Once the coding was completed, statistical analyses such as independent sample t-tests were performed. Although our sample size is relatively small for parametric tests, independent sample t tests produce error rates close to 5% and are adequately powered when effect sizes are large [38]. As our analyses produced large effect sizes, we feel the use of independent samples t tests in this study is justified.

## Results

A total of 315 memory descriptions were gathered. In 75 trials, most of which were cued by negative emotional words such as *sad, guilt* and *regretful,* participants failed to retrieve a memory. The success of recall was comparable; depressed participants retrieved 156 memories, whereas non-depressed retrieved 159 memories.

### Retrieval time

Retrieval time for all memories produced by each participant (up to 15 memories) was averaged. Since the retrieval times for both groups were approximately normally distributed with no outliers, the data were qualified for independent-sample *t*-test. The results revealed that depressed participants (M = 10.37 seconds, SD = 1.61) were faster in recollection than non-depressed participants (M = 13.55 seconds, SD = 2.67), *t*(24) = 3.67, p < 0.01, Cohen’s *d* = 1.50.

### Specificity of recollection

Memory descriptions were examined for six types of knowledge as mentioned earlier and counted for quantitative analysis. Consider the following two memories, the first retrieved by a depressed participant and the second by a non-depressed participant in which different types of knowledge/memory elements have been marked (within parentheses).

*Watching a movie in Malaysian cinema is cheaper than other countries (OTH). However, I rarely go to the cinema (GE). I only go four or five times a year (GE)* (retrieved by a depressed participant in response to the cue, CINEMA)

*It happened during my first year in Cairo…it was in 2001 (LTP). I had just finished my first year final exam (GE). I was not brave enough to see my exam results because I felt it was bad (MIX). I thought I was the most stupid student in Cairo (TH), I mean…in my class (TH). But suddenly, my friend called me. Her name is X. Her house is near em…What’s the name…em…Near Y factory…(ESK). I’ve forgotten the name of that district (OTH). She is one of my best friends (OTH). She said “you should see your exam results”, I replied “I do not want to see it” (ESK). I thought that I’ve definitely failed (TH). She said “No! You should see your results…you should come to the campus” (ESK). She told me that I passed with a very good grade, number 11 from the top (ESK)* (retrieved by a non-depressed participant in response to the cue, TELEPHONE)

As can be seen in the first memory, two *GE* and one *OTR* knowledge element were retrieved. In the second memory, one *LTP*, one *GE*, four *ESK*, three *TH*, one *MIX*, and two *OTR* knowledge elements were reported. Elements were extracted in all 315 memories, then average scores were calculated for LTP, GE, ESK, TH, MIX and OTR for each participant. The means, standard deviation scores and ranges for these memory elements are shown in Table 1. The distributions for memory elements for both samples approached normal distribution and there were no outliers, thus qualifying them for independent sample t-tests. The findings revealed significant differences between depressed and non-depressed for lifetime period, *t*(24) = 3.77, *p* < 0.01, *d* = 1.54, general event, *t*(24) = 2.11, *p* < 0.05, *d* = 0.86, event specific knowledge, *t*(24) = 6.44, *p* < 0.01, *d* = 2.63 and thought, *t*(24) = 3.66, *p* < 0.01, *d* = 1.49. Depressed participants reported less lifetime period knowledge, general event knowledge and event specific knowledge than the non-depressed participants. However, depressed participants were more likely to report thought components than the non-depressed participants. Cohen’s *d* values indicate large effects for all four memory elements.

**Table 1:** **Mean (and standard deviation) scores for depressed and non-depressed participants on memory elements, emotional valence, and retrieval sequence**

	**Memory count**	**Depressed**		**Non-depressed**	
***Memory elements***		**Mean ± SD**	**Range**	**Mean ± SD**	**Range**
ESK		0.40 ± 0.27	0.10-1.08	1.40 ± 0.49	0.42-2.62
GE		0.96 ± 0.41	0.23-1.82	1.25 ± 0.29	0.83-1.69
LTP	315	0.34 ± 0.14	0.00-0.55	0.53 ± 0.11	0.39-0.75
TH		0.56 ± 0.21	0.27-1.00	0.27 ± 0.20	0.08-0.73
MIX		0.37 ± 0.17	0.08-0.62	0.45 ± 0.23	0.17-0.91
OTR		0.41 ± 0.17	0.15-0.77	0.41 ± 0.16	0.15-0.75
*Emotional valence*					
Positive		0.14 ± 0.06	0.00-0.23	0.33 ± 0.07	0.18-0.46
Negative	315	0.52 ± 0.12	0.33-0.73	0.42 ± 0.11	0.18-0.58
Neutral		0.35 ± 0.12	0.09-0.50	0.25 ± 0.09	0.08-0.39
*Retrieval sequence*					
LTP → GE → ESK		0.10 ± 0.11	0.00-0.33	0.36 ± 0.18	0.09-0.73
LTP → ESK		0.06 ± 0.11	0.00-0.33	0.09 ± 0.25	0.00-0.90
LTP → GE	206	0.39 ± 0.16	0.00-0.60	0.16 ± 0.12	0.00-0.30
GE → ESK		0.32 ± 0.26	0.00-1.00	0.34 ± 0.14	0.13-0.58
GE → LTP		0.13 ± 0.12	0.00-0.33	0.17 ± 0.23	0.00-0.80

LTP = life time period knowledge; GE = general event knowledge; ESK = event specific knowledge.

To see whether the depressed and non-depressed participants varied in terms of the overall length of the memory descriptions, average scores for LTP, GE, ESK, TH, MIX and OTR for each participant calculated for earlier analyses were added. Independent sample *t*-test showed a significant difference, *t*(24) = 6.33, *p* < 0.01, *d* = 2.58 with depressed participants (M = 3.06, SD = 0.55) being less elaborative in their memory descriptions than the non-depressed participants (M = 4.33, SD = 0.47).

The memory content was also examined to see whether the depressed patients had a tendency to recall memories containing elements that were predominantly of positive or negative nature. In this case, the memory as a whole rather than its specific elements was considered. All memories were classified as either: positive (e.g., success, satisfaction), negative (e.g. personal failure, accidents), or a neutral memory (i.e. no positive or negative connotations). The second author and an external rater categorised those memories (96% agreement). Consider the following three memory protocols that were coded as positive, negative or neutral.

Positive: *In 2003, I was very happy on the day when I was discharged from the hospital. I felt satisfied because I didn’t like to stay there. I liked the doctors who discharged me from the ward. They gave me a lot of advice.* (Cue: HAPPY)

Negative: *It was when I went to the cinema with my husband during Depavalli celebration. Before we went to the movie, I saw my husband and my nephew sitting in front of our neighbour’s house which I didn’t like at all. I was so angry that I couldn’t watch the movie, rather I started sleeping. My nephew woke me up and said “aunt, please wake up and watch the movie”. I replied negatively. Then my husband came to me who I asked with despair why he sat in front of that house. Why…? We then came out of the cinema and went to a restaurant. They asked me to eat something but I also refused.* (Cue: CINEMA)

Neutral: *When I was about em…10 years old, I went to a cinema in Penang. I don’t remember the name of the cinema. The film was acted by P. Ramli and the title was Anakku Sazali.* (Cue: CINEMA)

The proportion of positive, negative and neutral memories was calculated for each participant. The data fulfilled the requirements of t-tests as they approached normal distribution with no outliers. The results showed depressed and non-depressed participants to vary in all three types of memories; positive, *t*(24) = 7.85, *p* < 0.01, *d* = 3.21, negative, *t*(24) = 2.21, *p* < 0.05, *d* = 0.90, and neutral, *t*(24) = 2.44, *p* < 0.05, *d* = 0.97. Depressed participants recalled fewer positive memories, but more negative and neutral memories compared to their non-depressed counterparts. Both groups retrieved more negative memories than positive or neutral memories (Table 1).

### Accessing the autobiographical knowledge base

To investigate whether the two groups differed in terms of knowledge sequence that they retrieved, 206 memories (77 from depressed and 129 from non-depressed participants) were selected from the total pool of 315 memories. These are the memories which contained various combinations of LTP, GE and ESK. Five knowledge sequences were identified (see below for examples).

1. LTP > GE > ESK sequence: *When my mother was still alive, I felt like killing her to relieve her pain as she was suffering from cancer (LTP). Um….before she passed away I made a mistake (GE). On one of the Mother’s Day…I really felt guilty when I saw that my mother was taking medicine on her own (ESK).* (Cue: GUILTY).
2. LTP > GE sequence: *I visited Washington DC in the USA for the first time in 1984 with my colleagues (LTP). The restaurant in which we ate our dinner on arrival was a Chinese restaurant (GE).* (Cue: RESTAURANT).
3. LTP > ESK sequence: *I’ve never had a birthday party in all my life because I was born in January…..in which month the school has always been open (LTP). In one occasion, I wanted to buy a dress and celebrate my birthday, but my mother refused to organize that and said “one day someone will celebrate for you” (ESK).* (Cue: HAPPY).
4. GE > ESK sequence: *After graduation, I was travelling with my friends (GE). One day we saw a child standing at the corner of a road – very sad and confused (ESK). We stopped and asked what happened, she replied that her mother was sick and they did not have money to visit a doctor (ESK). We quickly decided to visit her house; we saw her mother laying on the bed (ESK). We sent her to a doctor using our own money (ESK).* (Cue: SATISFACTION)
5. GE > LTP sequence: *When I don’t feel comfortable and cannot sleep I always lay down on a sofa (GE). I’ve been doing this since I was a child (LTP).* (Cue: CHAIR)

Memories were counted for all five knowledge sequences for each participant and converted to proportion values. The means, SDs and ranges of those proportion values are presented in Table 1. P-P plots were drawn to check if the distributions were skewed for all five knowledge sequences for both samples. The plots showed the distributions to be approximately normally distributed, fulfilling the requirement for independent sample t-tests. Moreover, there were no outliers detected in any of the distributions. The findings revealed that non-depressed participants retrieved significantly more memories that contained the sequence, LTP > GE > ESK compared to the depressed participants, *t*(24) = 4.43, *p* < 0.01, *d* = 1.81. However, depressed participants retrieved more knowledge sequences of the form LTP > GE compared to their non-depressed counterparts, *t*(22.73)^a^ = 4.05, *p* < 0.01, *d* = 1.70 [39]. No other differences in retrieval sequences were observed. In order to see if depressed participants retrieved more overgeneral memories, two knowledge sequences, LTP > GE and GE > LTP were examined. In these two sequences, the participants only reported general and abstract knowledge rather than specific details of a particular event. As they represented overgeneral memories, LTP > GE and GE > LTP are combined, which revealed that depressed participants recalled more overgeneral memories (51%) than their non-depressed counterparts (33%).

## Discussion

A substantial amount of research suggests that depressed individuals are impaired in their ability to recall specific autobiographical memories, showing a tendency to be overgeneral in their recall [2-4,1]. According to Conway and Pleydell-Pearce, individuals access specific autobiographical memories utilising a hierarchical search strategy, accessing life time period knowledge first, then general event knowledge and finally event specific knowledge [18]. While this holds true for non-depressed individuals [32], it has been proposed that depressed individuals terminate their search for specific memories early in this hierarchical search. While depressed individuals have previously been shown to report more general level information than non-depressed individuals, to date the sequence in which depressed individuals access autobiographical memories has not been directly tested. This study aimed to examine the search strategies used by depressed and non-depressed individuals to determine whether depressed individuals adhered to the sequential hierarchical search as proposed by Conway and Pleydell-Pearce [18].

**Figure 1:**
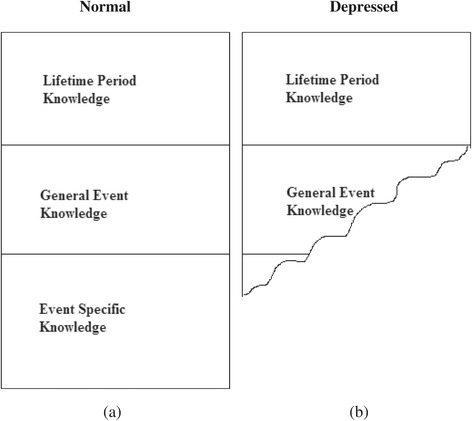
**Schematic representation of knowledge search sequence by (a) non-depressed (specific memory) and (b) depressed participants (overgeneral memory).**

Consistent with previous studies, our results revealed that depressed participants retrieved fewer memories overall, and their descriptions of events were shorter than the descriptions produced by the non-depressed participants. The results also revealed that depressed participants were less likely to retrieve specific event knowledge, and that memories were less likely to be positive in nature. In a novel result for this field, and offering support for Conway and Pleydell-Pearce’s model, the depressed participants also showed impairments in their ability to move sequentially through the hierarchy of the knowledge base in order to construct an autobiographical memory. The non-depressed participants retrieved autobiographical memories by first accessing the most abstract knowledge (lifetime periods) followed by less abstract general event knowledge and then specific event knowledge (LTP → GE → ESK). However, while depressed participants began their search by accessing lifetime period knowledge, their search was more likely to terminate at general event knowledge from the intermediate level of the hierarchy (LTP → GE; Figure 1). That depressed participants recalled fewer, less detailed memories might also explain why depressed participants were faster to recall memories in response to cue words.

Impaired executive control and rumination may explain the tendency for depressed participants to terminate their search early, and thus report less specific autobiographical memories, a finding which mirrors previous findings in this field [3,40,4,1,5]. Intrusive cognitions about past events are important determinants of overgeneral recall as they consume working memory capacity [40]. Consequently, although beginning their search by accessing lifetime period knowledge and general event knowledge, limited executive control capacity inhibits the ability to continue the search for event specific knowledge. Intrusions also promote rumination about the past and one’s self, and as previously mentioned, result in more abstract self-referent memories, rather than specific memories appropriate to the cue word [29,30]. As the depressed participants produced more thought elements in their memory descriptions than non-depressed participants it seems plausible that such thoughts would interrupt the retrieval of specific memories.

Although depressed participants reported fewer positive memories relative to the non-depressed group, both depressed and non-depressed participants were more likely to recall negative than positive memories. Although older adults tend to show a bias toward recalling positive events, numerous studies have shown that younger adults demonstrate a negativity bias [41]. Although spanning a wide age range, the majority of our participants were relatively young, thus a negativity bias is not unexpected. Further research would benefit from an examination of how the hierarchical search for autobiographical memories is affected by aging.

While further understanding of the search strategy used by depressed participants to recall autobiographical memories is important, the results of the study must be viewed in light of the study’s limitations. First, the relatively small sample limits the generalisability of the findings. Second, the two groups were recruited based on the presence or absence of major depressive disorder. Yet the two samples may also differ on other variables central to the relationship under investigation. Consideration of individual difference factors, such as rumination, would be informative in future studies examining the sequencing of autobiographical recall. Third, this study did not examine whether positive and negative memories were recalled in a similar fashion across the two groups. Previous research has noted differences in retrieval time for positive and negative memories [8], however examination of whether positive or negative memories are retrieved following the same hierarchical sequence would inform future research in this area. Further, examination of how attention, rumination and affect regulation may differentially affect the search sequence would also be informative. It is possible, for example, that rumination impairs the ability to access lifetime period knowledge, limited working memory capacity impedes access to general event information and that functional avoidance of negative affect might limit access to event specific knowledge. To our knowledge, the differential effect of these three processes on the hierarchical search sequence has not been directly examined. Finally, although our results suggest that depressed individuals are impaired in their ability to systematically search through the knowledge hierarchy to access specific autobiographical memories, it is also possible that depressed participants gave up their search at an intermediate level as they found it difficult or tiring to proceed further. Given the length of the testing session, and the fact that fatigue is commonly associated with depression, future studies should attempt to control for such a possibility.

## Conclusions

Despite the above limitations, the current study provides preliminary support for the notion that depressed individuals do attempt to access specific autobiographical memories following the hierarchical search strategy outlined by Conway and Pleydell-Pearce [18]. However, these participants are more likely to terminate the search at an intermediate level (general event knowledge), thus producing overgeneral memories in response to a request for specific autobiographical memories. Confirmation that the same hierarchical search sequence is attempted in both depressed and non-depressed individuals opens the way for future research to examine specific elements of the sequence in both participant groups.

### Endnote

^a^The assumption of homogeneity of variance was violated for this analysis, thus Satterthwaite [39] approximate degrees of freedom were used.
